# Omission of intraoperative drain placement during robotic partial nephrectomy and robotic radical prostatectomy is safe: an analysis of 18,000 patients

**DOI:** 10.1007/s00345-024-05320-7

**Published:** 2024-10-29

**Authors:** John Pfail, Jake Drobner, Alain Kaldany, Kevin Chua, Benjamin Lichtbroun, Rachel Passarelli, Hiren Patel, Arnav Srivastava, David Golombos, Thomas L. Jang, Vignesh T. Packiam, Saum Ghodoussipour

**Affiliations:** 1 Section of Urologic Oncology, Rutgers Cancer Institute and Rutgers Robert Wood Johnson Medical School, New Brunswick, NJ USA; 2 Department of Urology, University of California at San Francisco, San Francisco, CA USA; 3 Division of Urologic Oncology, University of Michigan Rogel Cancer Center, Ann Arbor, MI USA

**Keywords:** Urologic oncology, Intraperitoneal drain, Postoperative complications, Robotic prostatectomy, Robotic partial nephrectomy

## Abstract

**Purpose:**

Placement of a drain during robotic assisted partial nephrectomy (RAPN) and robotic assisted radical prostatectomy (RARP) is standard practice for many urologists and can aid in assessment and management of complications such as urine leak, lymphocele, or bleeding. However, drain placement can cause discomfort and delay patient discharge, with questionable benefit. We aim to assess the correlation between drain placement with post operative complications.

**Methods:**

The NSQIP targeted database was queried for patients who underwent RAPN or RARP from 2019 to 2021. Our primary outcomes included 30-day complication rates stratified by intraoperative drain placement. Secondary outcomes included procedure-specific complications, length of stay (LOS), and readmissions. Multivariable regression analyses, with Bonferroni correction, were performed for each post-operative complication.

**Results:**

We identified 4738 and 13,948 patients who underwent RAPN and RARP, respectively. Drains were not placed in 2258 (47.7%) and 6700 (48%) patients, respectively. On adjusted multivariable analysis in the RAPN cohort, omission of drain placement was associated with decreased LOS (β -0.45; 99.58% CI [-0.59, -0.32]) but no difference in overall complication rates. After adjusted analysis in the RARP cohort, omission of drain placement was associated with decreased risk of any complication (OR 0.73 [0.62–0.87]), infectious complication (OR 0.66 [0.49–0.89]), and LOS (β -0.30 [-0.37, -0.24]).

**Conclusions:**

Using a large contemporary database, this study demonstrates that omission of drains during RAPN and RARP was safe without increased risk of postoperative complications. Despite inherent selection bias in this cohort, our data suggests that routine drain placement is not necessary for these procedures.

**Supplementary Information:**

The online version contains supplementary material available at 10.1007/s00345-024-05320-7.

## Highlights

Foregoing drains can be safe in select patients undergoing robotic prostatectomy and partial nephrectomy.

Foregoing drain placement did not increase the risk of overall complications or readmission.

Drain placement was associated with increased hospital length of stay.

**Supplementary Information:**

The online version contains supplementary material available at 10.1007/s00345-024-05320-7.

## Introduction

Abdominal drains are routinely placed for patients undergoing surgical management of urologic cancers. While the prophylactic placement of an intraabdominal or retroperitoneal drain is conventional for some surgeons, contemporary data suggest drains increase infection risk, patient morbidity, and hospital length of stay [1–4]. Rare drain-associated complications, such as intestinal hernia, vascular injury, and retraction into the abdomen, have also been described in the literature [5–9]. Moreover, drain placement may also increase patient discomfort. On the other hand, drains may help to prevent fluid collections, such as lymphoceles, and enable early detection of anastomotic leaks or bleeding events [10, 11].

Nevertheless, modern surgical pathways have enhanced recovery after surgery, and patients now can safely return home within 24 h after prostatectomy and nephrectomy [12, 13]. Thus, routine drain placement after urologic oncology procedures may become a barrier to timely discharge. Similarly, a recent systematic review and meta-analysis of randomized controlled trials in the colorectal literature showed that omission of pelvic and peritoneal drains was safe without increased risk of anastomotic leakage, mortality, wound infection, nor reoperation rates [14]. Furthermore, the colorectal Enhanced Recovery After Surgery (ERAS) guidelines also recommend against the routine placement of pelvic and peritoneal drains [15]. While no prospective randomized data exists within the field of urology, several studies have found that drain placement after robotic-assisted laparoscopic prostatectomy, even with pelvic lymph node dissection, confers no significant benefit with respect to postoperative complications [16–18]. Omitting drainage for partial nephrectomy has also demonstrated safe postoperative outcomes [19, 20]. A recent meta-analysis of drain placement in various urologic oncology surgeries recommended discontinuing routine drainage for prostatectomy and partial nephrectomy due to a reduction in overall complications for drainless procedures [21].

Given the limited evidence regarding the benefit of routine drain placement after major urologic oncology surgeries, the objective of this study is to utilize the National Surgical Quality Improvement Program (NSQIP) targeted database to compare 30-day complication rates and hospital outcomes in patients undergoing robotic partial nephrectomy (RAPN) and robotic prostatectomy (RARP) based on drain placement. We hypothesize that omission of drains will shorten hospital stays without effect on surgical complication rates.

## Methods

### Patient selection

We identified patients undergoing RAPN or RARP from the American College of Surgeons’ NSQIP database using the database’s targeted procedure-specific files for nephrectomy and prostatectomy. Patients were excluded if they were noted to have documented bleeding disorders or systemic inflammatory response syndrome (SIRS), sepsis, or septic shock within 48 h prior to surgery. All procedures were extracted from the 2019–2021 editions of the public use files, as these were the most recent versions available for our analysis. Since all the data in NSQIP is deidentified, this study was exempt from review at our institutional review board.

We identified the procedures patients underwent via common procedural terminology (CPT) codes. Procedures included from the nephrectomy file were partial nephrectomy (50543, 50240) and procedures included from the prostatectomy file were radical prostatectomy (55845, 55866, 55840, 55842, 55815, 55810, 55821, 55812, 55831, and 55801). All patients were stratified by drain status recorded in NSQIP (i.e., “Drain” vs. “Drainless” cohorts).

### Variables

We captured demographic variables – including age and sex – and clinical variables including comorbidity data. Pathologic T, N, and M staging was also extracted from the targeted datasets. Using this data, patients in the RAPN cohort were classified according to the American Joint Committee on Cancer (AJCC) staging. Patients in the RARP cohort were already classified into pathologic stage groups (organ-confined, locally advanced, and nodal metastases).

Operative characteristics included operative time and length of stay. 30-day postoperative complications were collected and grouped by category (wound, infectious, renal, gastrointestinal, or genitourinary). Wound complications included superficial, deep, organ space, and surgical site infections as well as wound dehiscence. Infectious complications included urinary tract infection (UTI), sepsis, and septic shock. Renal complications included renal failure, which was defined as a rise in creatinine of > 2 mg/dl from preoperative value. Gastrointestinal (GI) complications included postoperative ileus or rectal injury. Genitourinary (GU) complications included urine leak/fistula or ureteral obstruction. Additionally, procedure specific complications such as urine leak and lymphocele / fluid collection were also collected. Complications were categorized by either low or high grade (Supplemental Table 1). 30-day readmission rates and mortality were also investigated.

### Statistical analysis

Chi-squared test was used for nonparametric categorical variables and the Mann-Whitney U test was used for nonparametric continuous variables. Multivariable logistic regression was performed for 30-day complications. Additionally, a multivariable linear regression was performed for length of stay (LOS). In the regression analyses, any patient with missing data was not included in the analysis. Factors included in the multivariable analysis included those that were clinically relevant. On the final multivariable analysis for the RAPN cohort, covariates included age, year of surgery, antibiotic length, ASA, BMI, OR time, and AJCC stage. For the RARP cohort, covariates included age, year of surgery, antibiotic duration, ASA, BMI, prior pelvic radiation, prior pelvic surgery, number of nodes collected, OR time, and stage group.

Our primary outcome of interest was to assess overall postoperative complication rates following RAPN and RARP stratified by drain placement. Secondary outcomes of interest included grade of complication, procedure specific complications, hospital length of stay and 30-day mortality. Owing to testing of multiple hypotheses, we adjusted the *p* value for significance according to Bonferroni correction (0.05/12 = 0.0048), yielding a total type I error rate of 5% for each of the independent cohorts (RAPN and RARP). Following this correction, we presented 99.58% confidence intervals. All analyses were conducted using R (R foundation, Vienna) version 4.1.2.

## Results

We identified 4738 patients who underwent RAPN, all of whom had data available regarding drain placement. Drains were placed in 52.3% (2480/4738) of patients undergoing RAPN (Table 1). There were no significant differences in baseline characteristics among patients who had drains placed or omitted, apart from significantly longer antibiotic duration in those who had drains placed (91.3% vs. 93.6% <24 h, 7.66% vs. 5.76% 24–72 h, 1.05% vs. 0.66% >72 h, *p* = 0.01).

**Table 1:** Preoperative and operative characteristics for patients undergoing major urologic procedures, divided by procedure type, and further stratified by drain placement. Age and operating room (OR) time presented as median [interquartile range (IQR)]

	Robotic Partial Nephrectomy			Robotic Prostatectomy		
	Drain	Drainless	P-value	Drain	Drainless	P-value
Characteristic	*N* = 2480	*N* = 2258		*N* = 7248	*N* = 6700	
Age	61.0 [52.0;69.0]	61.0 [52.0;68.0]	0.231	64.0 [59.0;68.0]	64.0 [59.0;68.0]	**0.006**
Sex:			0.71			---
Female	890 (35.9%)	823 (36.4%)		---	---	
Male	1590 (64.1%)	1435 (63.6%)		7248 (100%)	6700 (100%)	
ASA:			0.394			0.088
<3	1109 (44.7%)	981 (43.4%)		4181 (57.7%)	3768 (56.2%)	
≥3	1371 (55.3%)	1277 (56.6%)		3067 (42.3%)	2932 (43.8%)	
Year of Operation:			< 0.001			**< 0.001**
2019	1060 (42.7%)	866 (38.4%)		3282 (45.3%)	2522 (37.6%)	
2020	891 (35.9%)	811 (35.9%)		2633 (36.3%)	2574 (38.4%)	
2021	529 (21.3%)	581 (25.7%)		1333 (18.4%)	1604 (23.9%)	
BMI	30.4 [26.7;34.8]	30.5 [26.9;35.1]	0.181	28.6 [25.8;31.9]	28.6 [25.9;31.7]	0.833
Diabetes	541 (21.8%)	500 (22.1%)	0.812	1035 (14.3%)	930 (13.9%)	0.514
Smoking History	420 (16.9%)	383 (17.0%)	1	732 (10.1%)	708 (10.6%)	0.379
COPD	69 (2.78%)	66 (2.92%)	0.839	126 (1.74%)	100 (1.49%)	0.279
HTN	1487 (60.0%)	1370 (60.7%)	0.637	3840 (53.0%)	3493 (52.1%)	0.326
Prior Chemotherapy	18 (0.73%)	16 (0.71%)	1	84 (1.16%)	58 (0.87%)	0.101
Prior Pelvic Radiation	---	---	---	65 (0.90%)	32 (0.48%)	**0.004**
Prior Pelvic Surgery	840 (33.9%)	742 (32.9%)	0.481	1284 (17.7%)	1061 (15.8%)	**0.003**
PLND Performed	---	---	---	6338 (87.4%)	5445 (81.3%)	**< 0.001**
Number of Nodes	---	---	---	6.00 [3.00;11.0]	5.00 [2.00;10.0]	**<0.001**
OR Time	186 [145;233]	162 [128;207]	**<0.001**	211 [171;259]	199 [161;244]	**<0.001**
AJCC Stage:			0.562			---
I	2274 (91.7%)	2063 (91.4%)		---	---	
II	44 (1.77%)	34 (1.51%)		---	---	
III-IV	162 (6.53%)	161 (7.13%)		---	---	
Stage Group:			---			**0.003**
Organ Confined	---	---		4181 (57.7%)	3687 (55.0%)	
Locally Advanced	---	---		2627 (36.2%)	2618 (39.1%)	
Nodal Metastasis	---	---		440 (6.07%)	395 (5.90%)	
Peri-Operative Antibiotics:			**0.011**			**< 0.001**
<24 h	2264 (91.3%)	2113 (93.6%)		6351 (87.6%)	6211 (92.7%)	
24–72 h	190 (7.66%)	130 (5.76%)		576 (7.95%)	369 (5.51%)	
>72 h	26 (1.05%)	15 (0.66%)		321 (4.43%)	120 (1.79%)	

We similarly identified 13,948 patients who underwent RARP, all of whom had data available regarding drain placement. Drains were placed in 52% (7248/13948) of patients who underwent RARP. Patients who underwent drain placement during RARP had higher rates of prior radiation (0.9% vs. 0.5%, *p* = 0.004) or pelvic surgery (17.7% vs. 15.8%, *p* = 0.003), more likely to have undergone pelvic lymph node dissection (PLND) (87.4% vs. 81.3%, *p* < 0.001), higher number of lymph nodes collected (median number of nodes: 6 vs. 5, *p* < 0.001), longer operative time (median OR time: 211 min vs. 199, *p* < 0.001), and less advanced pathological stage group (organ confined: 57.7% vs. 55%, *p* = 0.003) when compared to those who did not undergo drain placement.

**Fig. 1:**
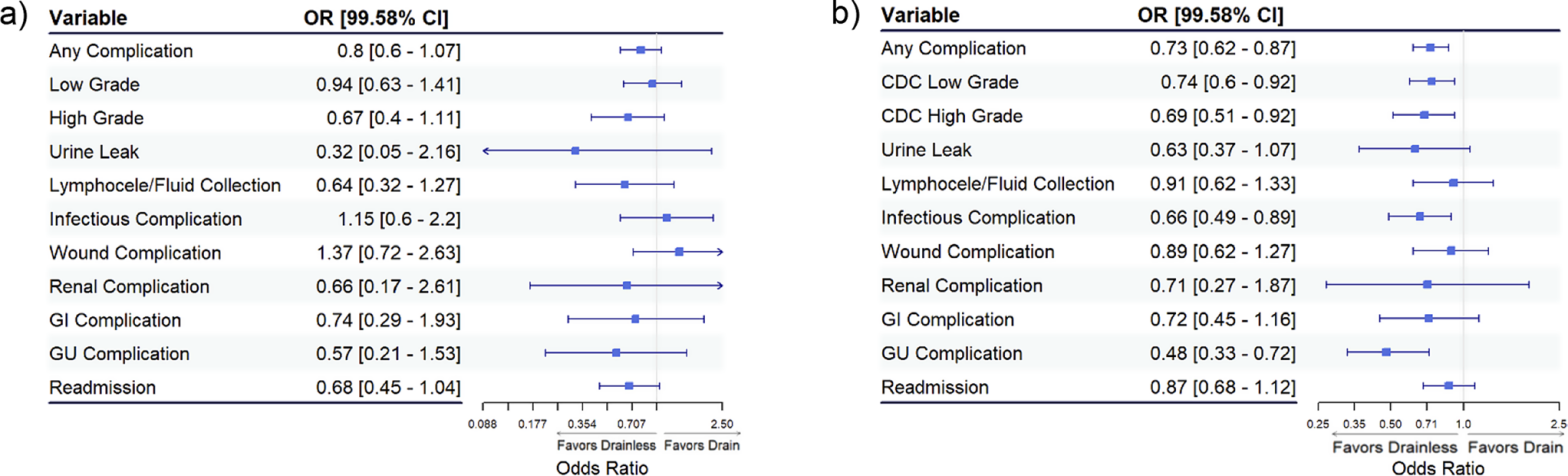
Odds ratios (OR) [99.58% CI] assessing the risk of complications associated with drain placement. (**a**) Robotic partial nephrectomy, covariates included age, year of surgery, antibiotic length, ASA, BMI, OR time, and AJCC stage. (**b**) Robotic prostatectomy, covariates included age, year of surgery, antibiotic duration, ASA, BMI, prior pelvic radiation, prior pelvic surgery, number of nodes collected, OR time, and stage group. *Reference = Drain placed*

On multivariable analysis, after Bonferroni adjustment, there was no statistically significant difference in the odds of any complication (OR 0.8; 99.58% CI [0.6–1.07]), high grade complication (OR 0.67; 99.58% CI [0.4–1.11]), or readmission (OR 0.68; 99.58% CI [0.45–1.04]) for patients who had omission of drains during RAPN compared to those who had drains placed (Fig. 1a). However, hospital length of stay (β -0.45; 99.58% CI [-0.59, -0.32]) was significantly lower for patients who had omission of drains during RAPN. Additionally, we found no difference in the number of patients who required postoperative drain placement or reoperation for a urine leak/fluid collection (1% vs. 0.7%, *p* = 0.322).

After Bonferroni adjustment, patients who had omission of drains during RARP experienced lower odds of any complication (OR 0.73; 99.58% CI [0.62–0.87]), as well as low (OR 0.74; 99.58% CI [0.6–0.92]), and high (OR 0.69; 99.58% CI [0.51–0.92]) grade complications. Additionally, patients who underwent drainless RARP experienced lower odds of infections (OR 0.66; 99.58% CI [0.49–0.89]) and GU specific complications (OR 0.48; 99.58% CI [0.33–0.72]) (Fig. 1b). Furthermore, hospital length of stay was significantly shorter in patients who underwent drainless RARP as well (β -0.30; 99.58% CI [-0.37, -0.24]). Lastly, there was no difference in the number of patients who required postoperative drain placement or reoperation for a urine leak/fluid collection (1.2% vs. 0.9%, *p* = 0.06). Complication rates are summarized in Table 2.

**Table 2:** Complication rates following robotic assisted partial nephrectomy and radical prostatectomy, stratified by drain placement

	Partial Nephrectomy			Radical Prostatectomy		
Complication:	Drain *N* = 2480	Drainless *N* = 2258	p	Drain *N* = 7248	Drainless *N* = 6700	p
Any Complication	270 (10.9%)	188 (8.33%)	0.003	845 (11.7%)	562 (8.39%)	< 0.001
Low Grade	126 (5.08%)	100 (4.43%)	0.325	497 (6.86%)	332 (4.96%)	< 0.001
High Grade	93 (3.75%)	54 (2.39%)	0.009	277 (3.82%)	166 (2.48%)	< 0.001
Urine Leak	11 (0.44%)	3 (0.13%)	0.089	92 (1.27%)	47 (0.70%)	0.001
Lymphocele / Fluid Collection	51 (2.06%)	28 (1.24%)	0.038	134 (1.85%)	105 (1.57%)	0.224
Infectious Complication	99 (3.99%)	93 (4.12%)	0.883	375 (5.17%)	255 (3.81%)	< 0.001
Wound Complication	39 (1.57%)	45 (1.99%)	0.325	154 (2.12%)	121 (1.81%)	0.196
Renal Complication	12 (0.48%)	7 (0.31%)	0.474	26 (0.36%)	14 (0.21%)	0.135
GI Complication	26 (1.05%)	16 (0.71%)	0.275	106 (1.46%)	60 (0.90%)	0.003
GU Complication	27 (1.09%)	13 (0.58%)	0.077	188 (2.59%)	80 (1.19%)	< 0.001
Readmission	132 (5.32%)	79 (3.50%)	0.003	335 (4.62%)	259 (3.87%)	0.03
Mortality 30d	7 (0.28%)	3 (0.13%)	0.349	6 (0.08%)	10 (0.15%)	0.364

In a subgroup analysis of laparoscopic procedures, which included 459 laparoscopic partial nephrectomies and 804 laparoscopic radical prostatectomies, no significant benefit of drain placement on postoperative outcomes was observed (Supplemental Fig. 1).

## Discussion

We used a contemporary cohort and found that roughly 50% of patients undergoing RAPN or RARP receive an intraoperative drain. The omission of drain placement was associated with a shorter length of stay in both RAPN and RARP, no difference in risk of complications after RAPN and was associated with a lower risk of complications after RARP. Additionally, patients who underwent either RAPN or RARP without drain placement had 1% risk of ultimately receiving a postoperative drain or reoperation for a urine leak/fluid collection.

Patients who underwent RAPN without intraoperative drain placement experienced shorter hospital length of stay (median [IQR] LOS 2 [1–3] days vs. 1 [1–2] days, *p* < 0.001) without difference in complication rates. While there were few differences in baseline characteristics amongst patients who underwent RAPN with or without drain placement, the majority of patients underwent surgery for stage 1 tumors, which is consistent with guideline recommended practice patterns [22]. Moreover, the drainless cohort had less use of postoperative antibiotics, which may have benefits not measured in this study. The dataset is limited in its ability to provide granular data on specific tumor characteristics, such as nephrometry score, that may influence a surgeon’s decision to place a drain [23], but the large number of patients in this national sample reflects real world practice patterns that suggest routine drain placement should be reconsidered.

Patients who underwent RARP without intraoperative drain placement were significantly less likely to have any complication (11.7% vs. 8.39%, *p* < 0.001), even after adjustment (OR 0.73; 99.58% CI [0.62–0.87]). While we did control for significant differences in the drain and drainless cohorts, including radiation history, longer operative times and extent of lymph node dissection, other unmeasured confounding variables may exist. Still, drainless RARP was associated with decreased hospital LOS on multivariable analysis (β -0.30; 99.58% CI [-0.37, -0.24]) and again, the rate of re-intervention or eventual placement of a drain in the drainless cohort was very low at 1.2%. These results again suggest that routine drain placement should be reconsidered, especially in patients where there is low concern for anastomotic leak.

Our study uses a large patient population to expand on the current data on drainless robotic urologic oncology surgeries. Beksac et al. recently used a multi-institutional database consisting of 904 patients who underwent robotic partial nephrectomy. In their study, 40% of patients underwent drainless robotic partial nephrectomy. Similar to our analysis, they concluded that drainless surgery was associated with shorter length of stay and no significant difference in overall complications or readmission rates [19]. A recent meta-analysis performed by Kowalewski et al. identified 1086 patients undergoing partial nephrectomy and 2520 patients undergoing radical prostatectomy. Their study revealed that drainless partial nephrectomy had similar rates of overall complications (OR: 0.99[0.65;1.51], *p* = 0.960) and re-intervention (OR: 1.16[0.31;4.38], *p* = 0.820). However, in their prostatectomy cohort, they found that drainless procedures were associated with a reduction in postoperative complications (OR: 0.62[0.44;0.87], *p* = 0.006) [21]. Another meta-analysis included 8447 patients undergoing partial nephrectomy and 1890 patients undergoing robotic radical prostatectomy. They found that patients undergoing robotic partial nephrectomy without drain had shorter length of hospital stay (mean difference: -0.84 days, 95% CI: -1.06 to -0.63; *P* < 0.001) and similar low-grade (*P* = 0.94) and high-grade (*P* = 0.31) complications, urinary leakage (*P* = 0.49), hemorrhage (*P* = 0.39), reintervention (*P* = 0.69), and readmission (*P* = 0.20) compared with routinely drained patients. They also found that patients undergoing drainless robotic prostatectomy had lower rate of postoperative ileus (OR 0.53, 95% CI: 0.38 to 0.74; *P* < 0.001), but similar low-grade (Clavien 1–2, *P* = 0.41) and high-grade (Clavien ≥ 3; *P* = 0.85) complications [24].

Given the unclear benefit of surgical drain placement in our analysis, there is a need to replace traditional dogmas with evidence-based practices, particularly as previous studies suggest that drain placement may negatively impact patient recovery. A single institutional analysis reported heightened postoperative pain with up to 24% of radical prostatectomy patients attributing postoperative pain to drain sites [25].

Our study has several limitations beyond its retrospective design and the limitations inherent to NSQIP. In particular, our data does not contain certain key intraoperative factors (such as a violation of the collecting system in a partial nephrectomy or transperitoneal vs. retroperitoneal approach) or other patient level variables (immunosuppressive disorders or peri-operative anti-coagulation requirements) that likely inform a surgeon’s decision. Second, the NSQIP database only reports 30-day complication and mortality rates. Thus, any complications related to a drainless procedure that occur after 30 days are unreportable. However, complications that may be identified by drain placement such as bleed, urine leak or lymphocele, are typically identified within 30 days. Furthermore, given the relatively limited number of nodes removed during RARP (median number of nodes: 6 vs. 5) in this dataset these findings may not be accurately extrapolated to patients undergoing extended PLND. Lastly, the placement of drains may be related to organizational or situational factors, such as the complexity of the case relative to the training stage of the surgeon, or hospital policy, and it is possible that this increased the number of patients who received drains. Due to the limitations within the dataset, we were unable to adjust for clustering. Randomized controlled trial data, including patient reported outcomes, is needed to directly compare outcomes and complications with respect to drain placement after urologic oncology surgeries in order to identify which patients will benefit most from drainage, while deferring the morbidity in those unlikely to benefit from drain placement. Furthermore, these analyses should include more detailed intraoperative data, particularly regarding the decision-making process behind the placement of a drain, such as difficult ureterovesical anastomosis, prostate size, nephrometry score or tumor complexity.

## Conclusion

Based on a large contemporary database, surgical drains are placed in about half of patients who undergo RAPN and RARP. Foregoing drain placement was not associated with increased complications after RAPN or RARP. Patients who underwent drainless RAPN or RARP experienced a shorter hospital length of stay with omission of drains. These data support the practice of omission of routine drain placement in patients undergoing RAPN and RARP.

## Electronic supplementary material

Supplementary Material 1

## Data Availability

Data analyzed is available via NSQIP data, which requires approval for access and analysis. As approval is necessary for data access, unsure if can submit for data availability.
