# Supplementary material for: Omission of intraoperative drain placement during robotic partial nephrectomy and robotic radical prostatectomy is safe: an analysis of 18,000 patients
Source: World J Urol. 2024 Oct 29;42(1):601. doi: 10.1007/s00345-024-05320-7 (PMC11522192; doi:10.1007/s00345-024-05320-7)
Supplement: Supplementary file 1 — Supplementary Material 1 [file 345_2024_5320_MOESM1_ESM.docx]

**Supplemental Tables and Figures:**

| Low Grade | Occurrences of Surgical Site Infection (SSI), deep incisional SSI, organ space SSI, fascial dehiscence, pneumonia, UTI, DVT, ileus (prolonged NPO/nasogastric tube), rectal injury identified postoperatively and not requiring intervention/surgery, C. diff, ureteral fistula (no operative intervention), ureteral obstruction (no operative intervention), lymphocele (no intervention), anastomotic leak (no intervention), progressive renal insufficiency |
| --- | --- |
|  | Unplanned reoperation, leak treated with intervention or reoperation, lymphocele with reoperation or intervention, ureteral obstruction requiring intervention |
| High Grade | Septic shock, PE, MI, cardiac arrest necessitating CPR, unplanned intubation, ventilator dependent >48 hours, acute renal failure necessitating dialysis, stroke |
|  | Patient death |

**Supplemental Table 1.**  Grading definitions for postoperative complications.


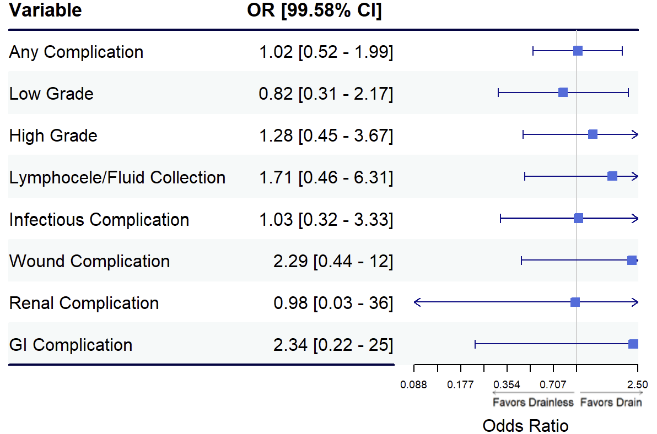

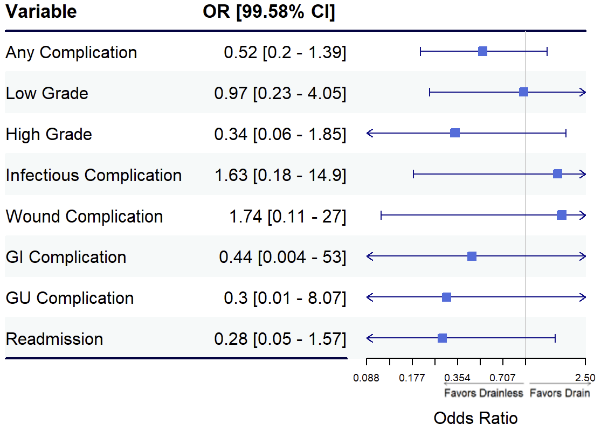


b)

a)

**Supplemental** **Figure 1.** Odds ratios (OR) [99.58% CI] assessing the risk of complications associated with drain placement. (a) Laparoscopic partial nephrectomy, covariates included age, year of surgery, antibiotic length, ASA, BMI, OR time, and AJCC stage. (b) Laparoscopic prostatectomy, covariates included age, year of surgery, antibiotic duration, ASA, BMI, prior pelvic radiation, prior pelvic surgery, number of nodes collected, OR time, and stage group. *Reference = Drain placed.*
